# Supplementary material for: Genomic Hatchery Introgression in Brown Trout (Salmo trutta L.): Development of a Diagnostic SNP Panel for Monitoring the Impacted Mediterranean Rivers
Source: Genes (Basel). 2022 Jan 28;13(2):255. doi: 10.3390/genes13020255 (PMC8872556; doi:10.3390/genes13020255)
Supplement: Supplementary file 1 [file genes-13-00255-s001.zip › SupMat_Casanovas/Figures S1-S11.pdf]

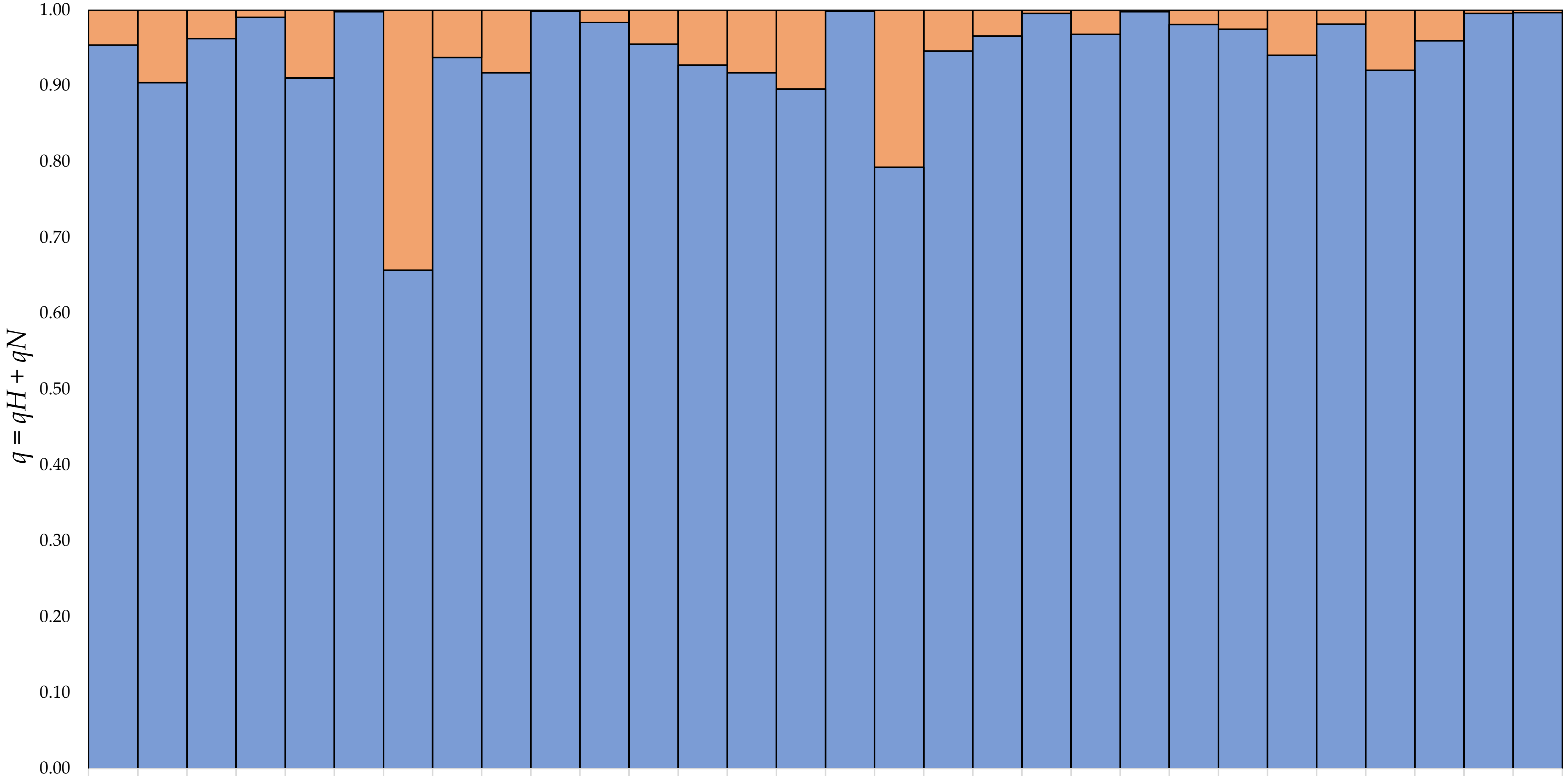

	631	632	633	634	635	636	637	638	639	640	641	642	643	644	645
qH	0.047	0.096	0.038	0.010	0.090	0.002	0.343	0.063	0.083	0.002	0.016	0.045	0.073	0.083	0.104
qN	0.954	0.904	0.962	0.990	0.910	0.998	0.657	0.937	0.917	0.998	0.984	0.955	0.927	0.917	0.896

	646	647	648	649	650	651	652	653	654	655	656	657	658	659	660
qH	0.002	0.207	0.054	0.035	0.005	0.032	0.002	0.019	0.026	0.060	0.019	0.079	0.041	0.004	0.004
qN	0.998	0.793	0.946	0.965	0.995	0.968	0.998	0.981	0.974	0.940	0.981	0.921	0.959	0.996	0.996

QU16

Figure S1. STRUCTURE plot for the individual admixture coefficient values (q) at QU16 using the 19 diagnostic SNPs genotyped with MassARRAY approach. Each individual is represented as a vertical bar partitioned into segments according to the proportion of the genome belonging to each of the two clusters (K) identified (Hatchery (H) and Native (N), respectively) by STRUCTURE.

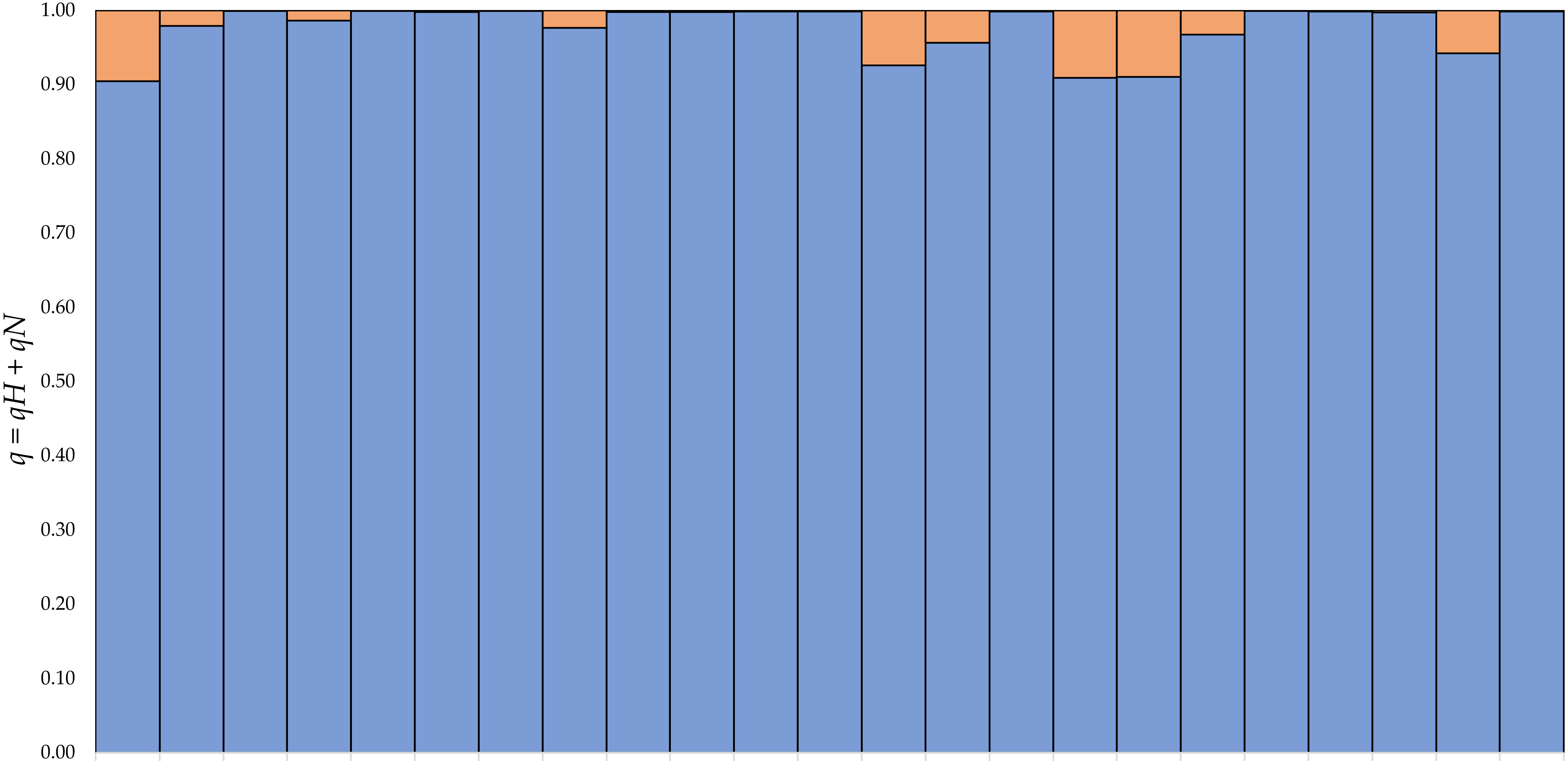

	661	662	663	664	665	666	667	668	669	670	671	672	673	675	676	677	678	679	680	681	682	683	684
qH	0.095	0.021	0.001	0.013	0.001	0.002	0.001	0.023	0.002	0.002	0.002	0.002	0.074	0.043	0.002	0.091	0.089	0.032	0.001	0.002	0.003	0.058	0.002
qN	0.905	0.979	0.999	0.987	0.999	0.998	0.999	0.977	0.998	0.998	0.998	0.998	0.926	0.957	0.998	0.909	0.911	0.968	0.999	0.998	0.997	0.942	0.998

ME16

Figure S2. STRUCTURE plot for the individual admixture coefficient values (*q*) at ME16 using the 19 diagnostic SNPs genotyped with MassARRAY approach. Each individual is represented as a vertical bar partitioned into segments according to the proportion of the genome belonging to each of the two clusters (K) identified (Hatchery (H) and Native (N), respectively) by STRUCTURE.

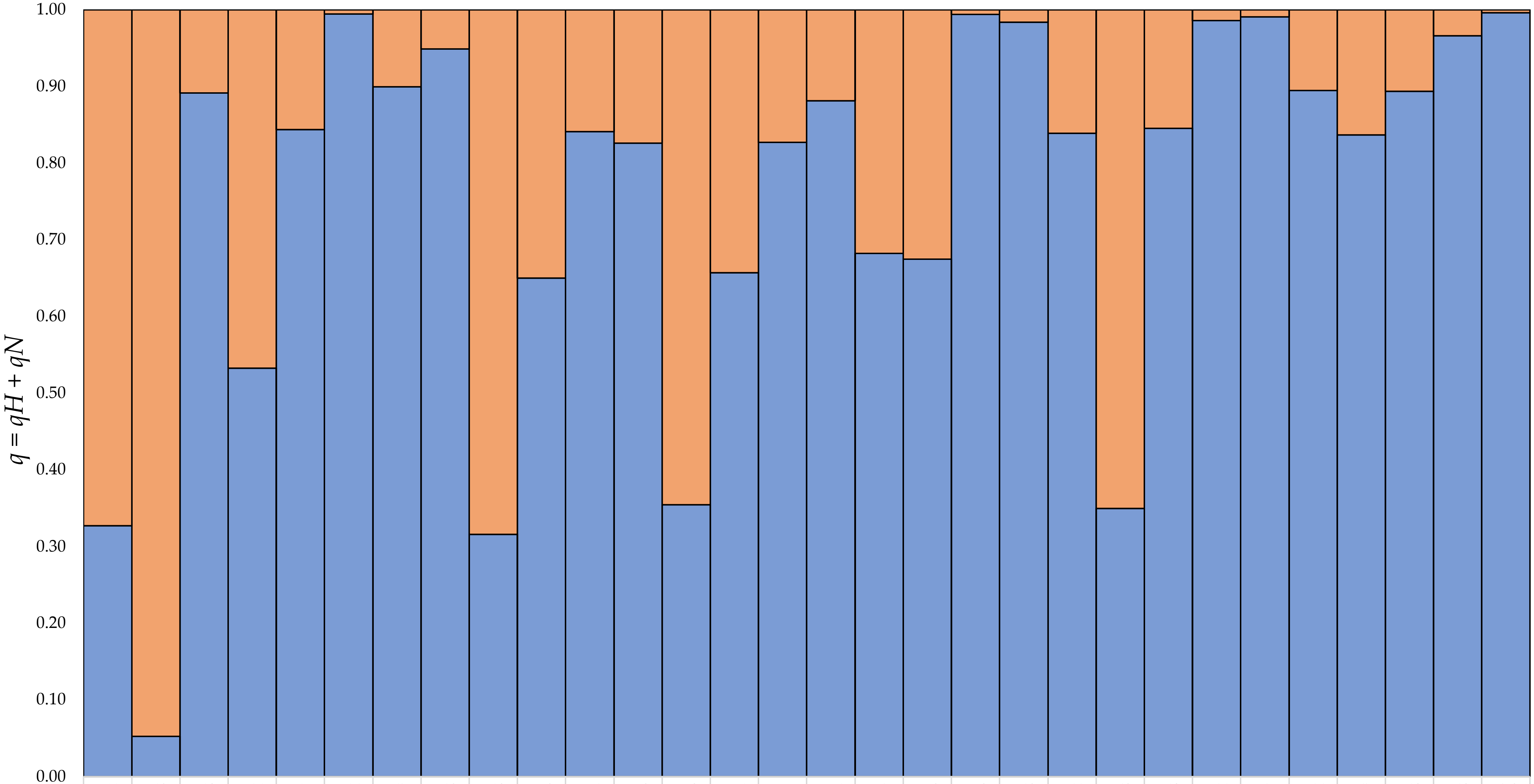

	541	542	543	544	545	546	547	548	549	550	551	552	553	554	555	556	557	558	559	560	561	562	563	564	565	566	567	568	569	570
qH	0.673	0.947	0.109	0.467	0.156	0.005	0.100	0.051	0.684	0.350	0.159	0.174	0.646	0.343	0.173	0.119	0.318	0.325	0.006	0.016	0.161	0.650	0.155	0.014	0.009	0.105	0.163	0.106	0.034	0.004
qN	0.327	0.053	0.891	0.533	0.844	0.995	0.900	0.949	0.316	0.650	0.841	0.826	0.354	0.657	0.827	0.881	0.682	0.675	0.994	0.984	0.839	0.350	0.845	0.986	0.991	0.895	0.837	0.894	0.966	0.996

PR16

Figure S3. STRUCTURE plot for the individual admixture coefficient values (q) at PR16 using the 19 diagnostic SNPs genotyped with MassARRAY approach. Each individual is represented as a vertical bar partitioned into segments according to the proportion of the genome belonging to each of the two clusters (K) identified (Hatchery (H) and Native (N), respectively) by STRUCTURE.

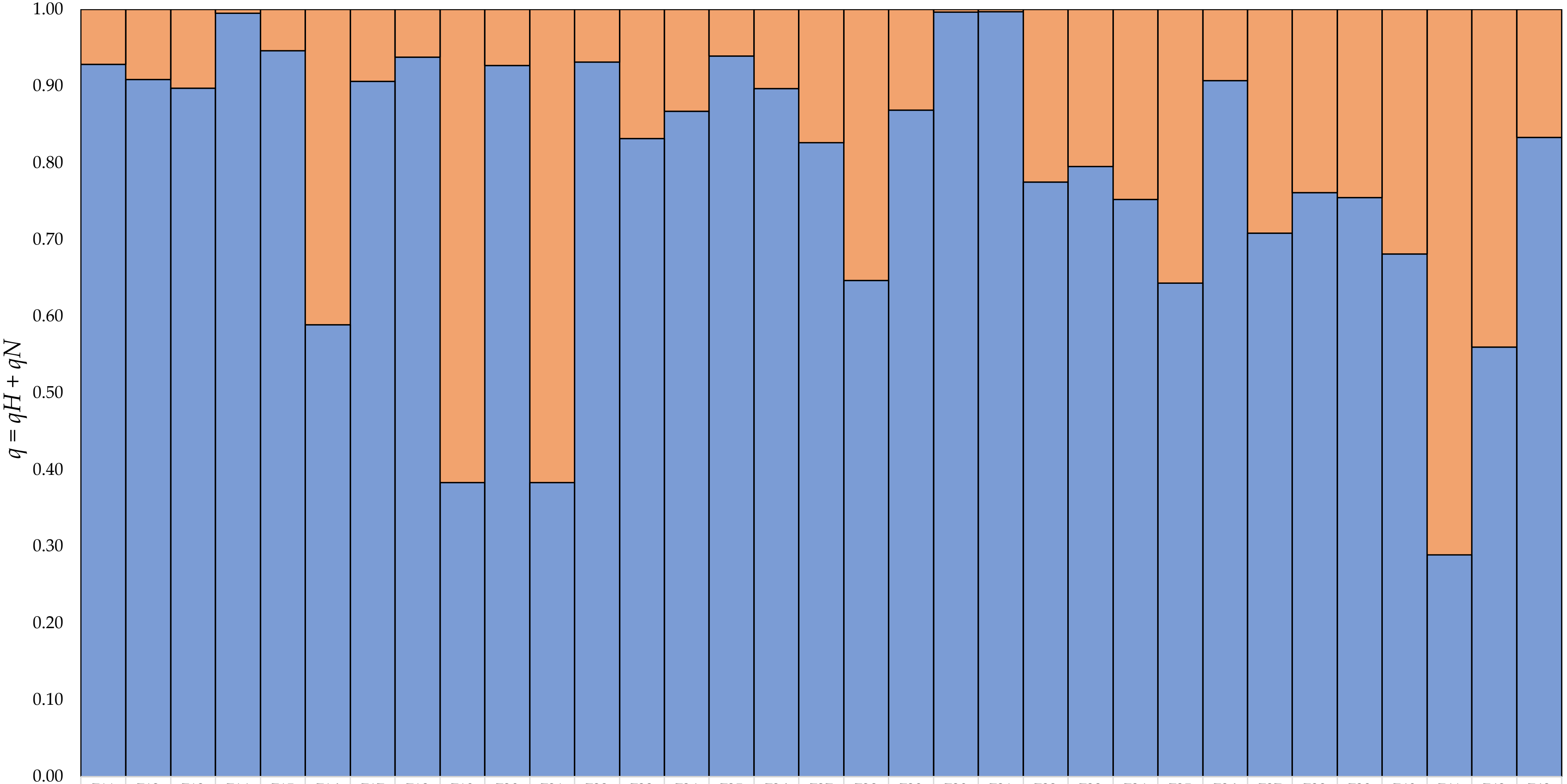

	711	712	713	714	715	716	717	718	719	720	721	722	723	724	725	726	727
qH	0.072	0.091	0.103	0.005	0.054	0.411	0.094	0.062	0.617	0.073	0.617	0.069	0.169	0.133	0.061	0.103	0.174
qN	0.928	0.909	0.897	0.995	0.946	0.589	0.906	0.938	0.383	0.927	0.383	0.931	0.831	0.867	0.939	0.897	0.826

	728	729	730	731	732	733	734	735	736	737	738	739	740	741	742	743
qH	0.353	0.131	0.004	0.003	0.225	0.205	0.248	0.357	0.093	0.292	0.239	0.245	0.319	0.711	0.440	0.167
qN	0.647	0.869	0.996	0.997	0.775	0.795	0.752	0.643	0.907	0.708	0.761	0.755	0.681	0.289	0.560	0.833

Figure S4. STRUCTURE plot for the individual admixture coefficient values (q) at MA17 using the 19 diagnostic SNPs genotyped with MassARRAY approach. Each individual is represented as a vertical bar partitioned into segments according to the proportion of the genome belonging to each of the two clusters (K) identified (Hatchery (H) and Native (N), respectively) by STRUCTURE.

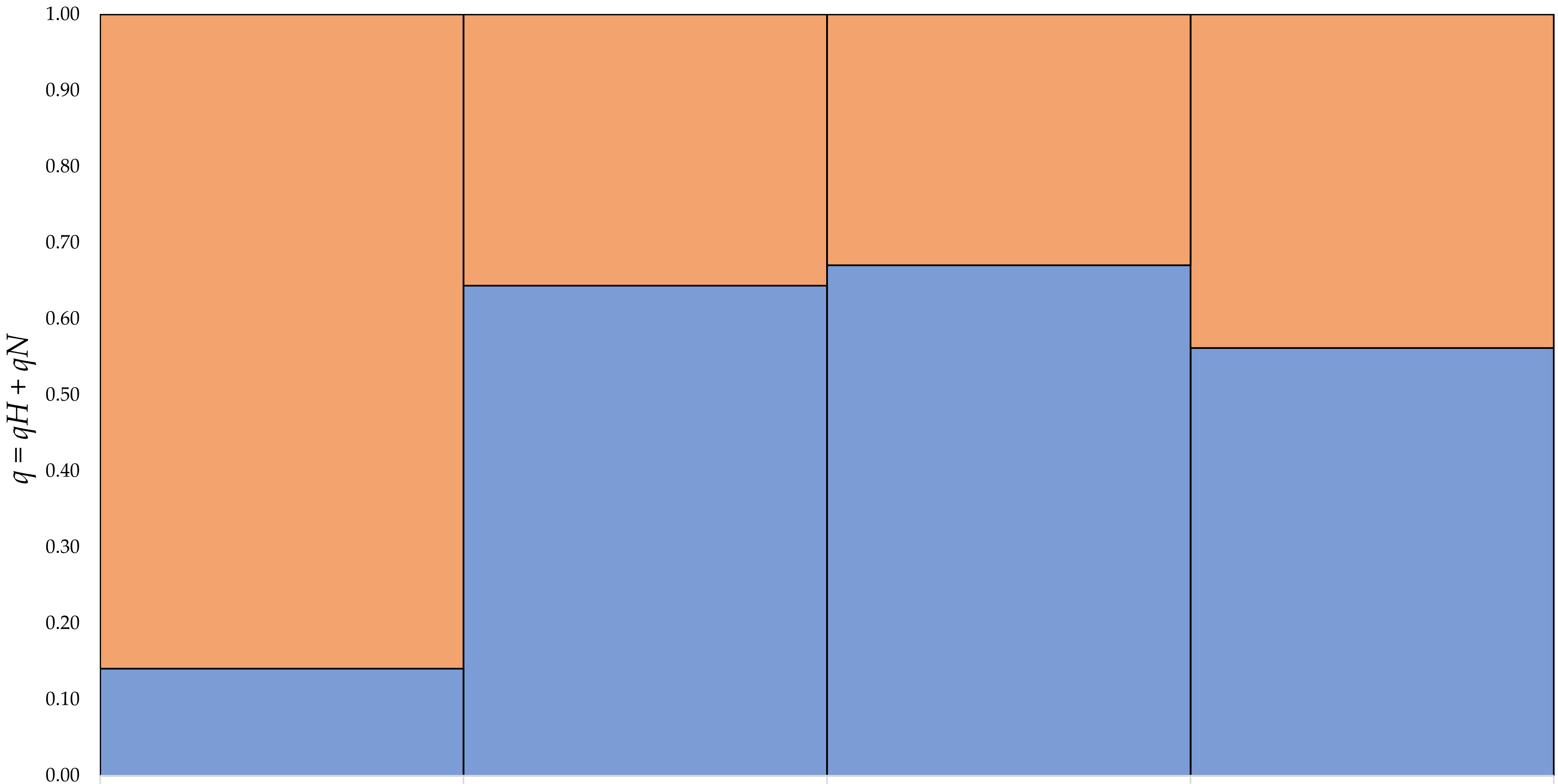

	687	688	689	690
qH	0.859	0.356	0.329	0.438
qN	0.141	0.644	0.671	0.562

TS18

Figure S5. STRUCTURE plot for the individual admixture coefficient values (*q*) at TS18 using the 19 diagnostic SNPs genotyped with MassARRAY approach. Each individual is represented as a vertical bar partitioned into segments according to the proportion of the genome belonging to each of the two clusters (K) identified (Hatchery (H) and Native (N), respectively) by STRUCTURE.

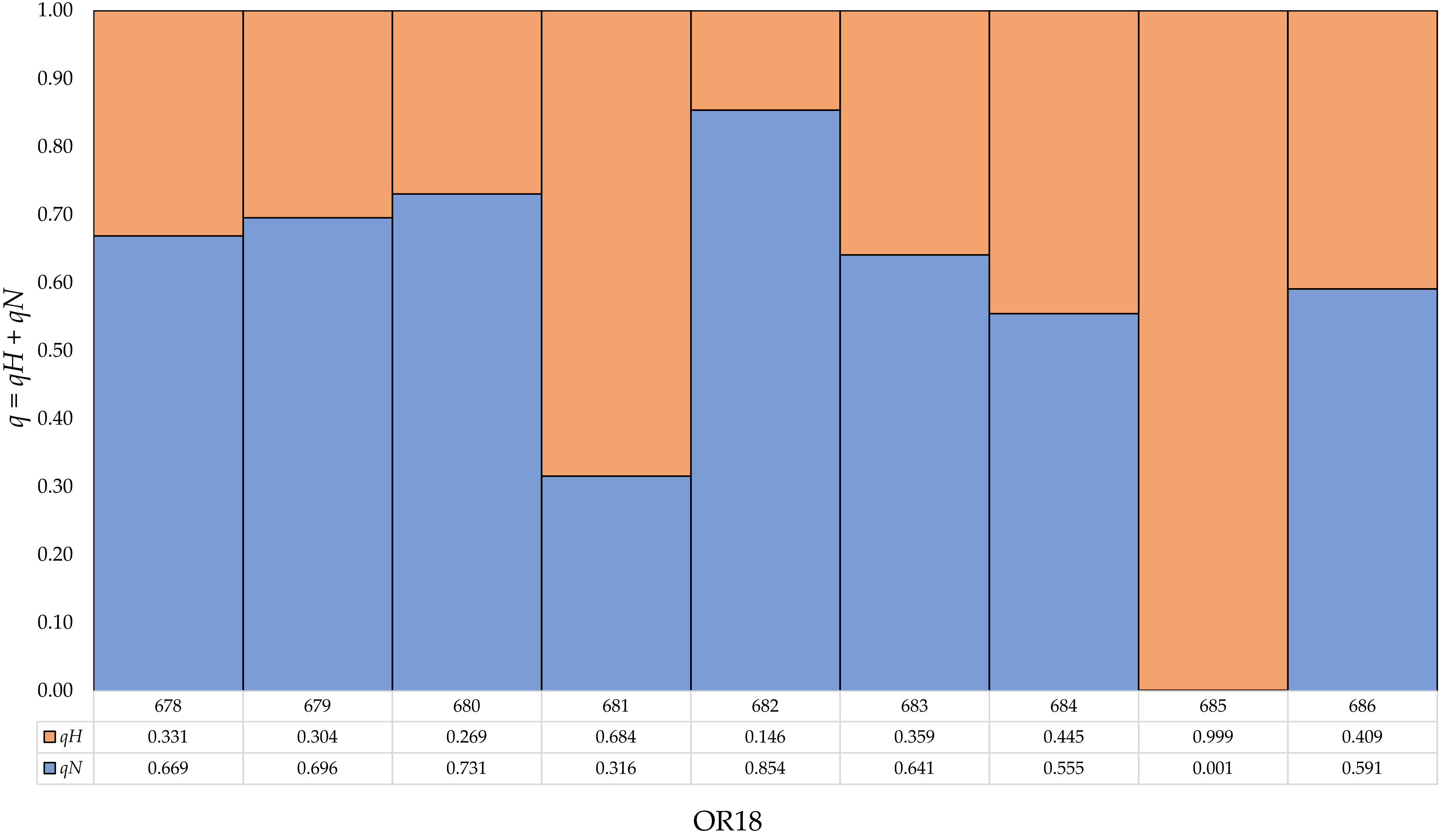

	678	679	680	681	682	683	684	685	686
qH	0.331	0.304	0.269	0.684	0.146	0.359	0.445	0.999	0.409
qN	0.669	0.696	0.731	0.316	0.854	0.641	0.555	0.001	0.591

Figure S6. STRUCTURE plot for the individual admixture coefficient values (q) at OR18 using the 19 diagnostic SNPs genotyped with MassARRAY approach. Each individual is represented as a vertical bar partitioned into segments according to the proportion of the genome belonging to each of the two clusters (K) identified (Hatchery (H) and Native (N), respectively) by STRUCTURE.

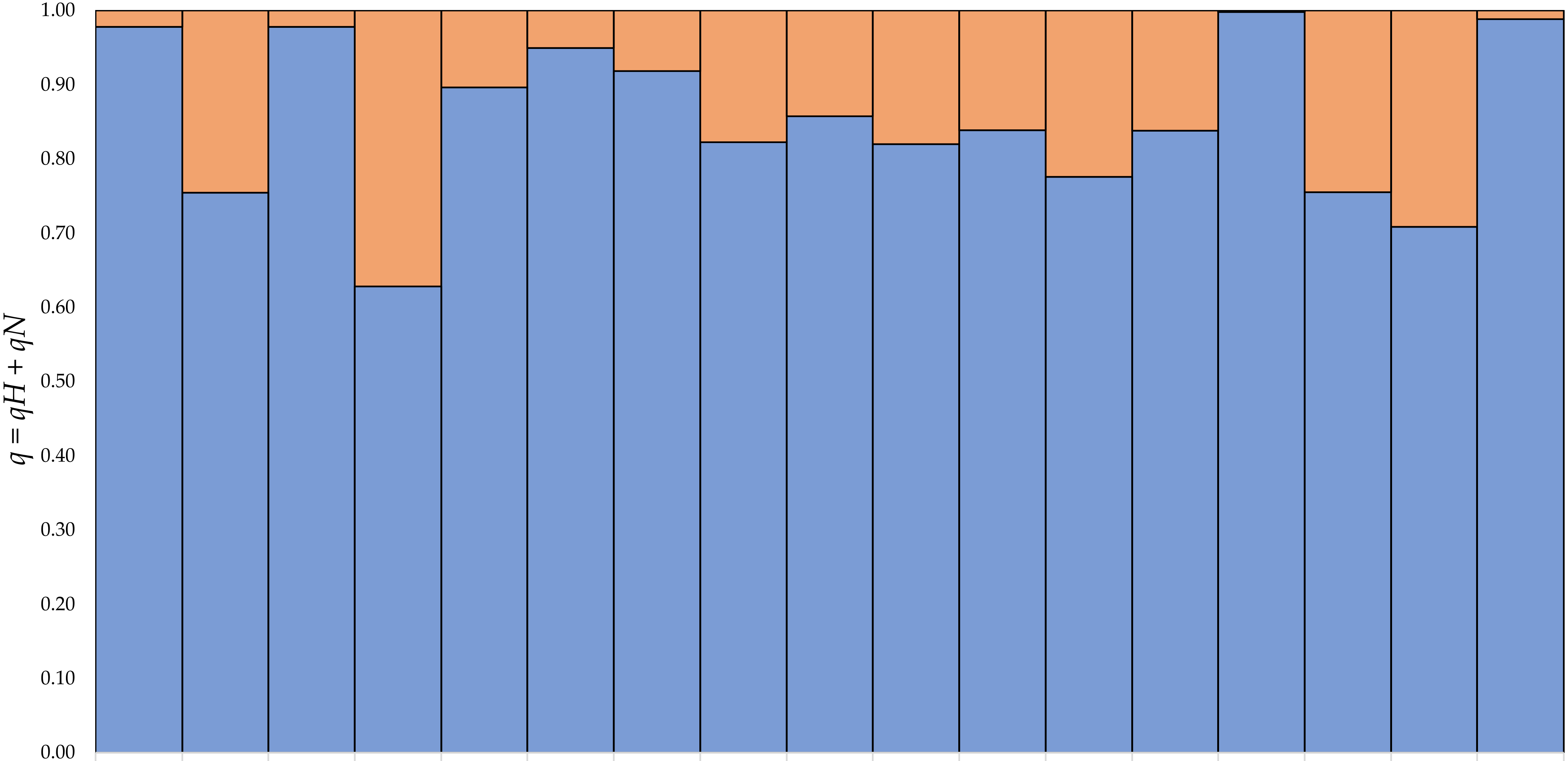

	354	355	356	357	358	359	360	361	362	363	364	365	366	367	368	369	370
qH	0.022	0.245	0.022	0.371	0.104	0.050	0.081	0.177	0.143	0.180	0.161	0.224	0.162	0.002	0.245	0.291	0.012
qN	0.978	0.755	0.978	0.629	0.896	0.950	0.919	0.823	0.857	0.820	0.839	0.776	0.838	0.998	0.755	0.709	0.988

Figure S7. STRUCTURE plot for the individual admixture coefficient values (q) at CA18 using the 19 diagnostic SNPs genotyped with MassARRAY approach. Each individual is represented as a vertical bar partitioned into segments according to the proportion of the genome belonging to each of the two clusters (K) identified (Hatchery (H) and Native (N), respectively) by STRUCTURE.

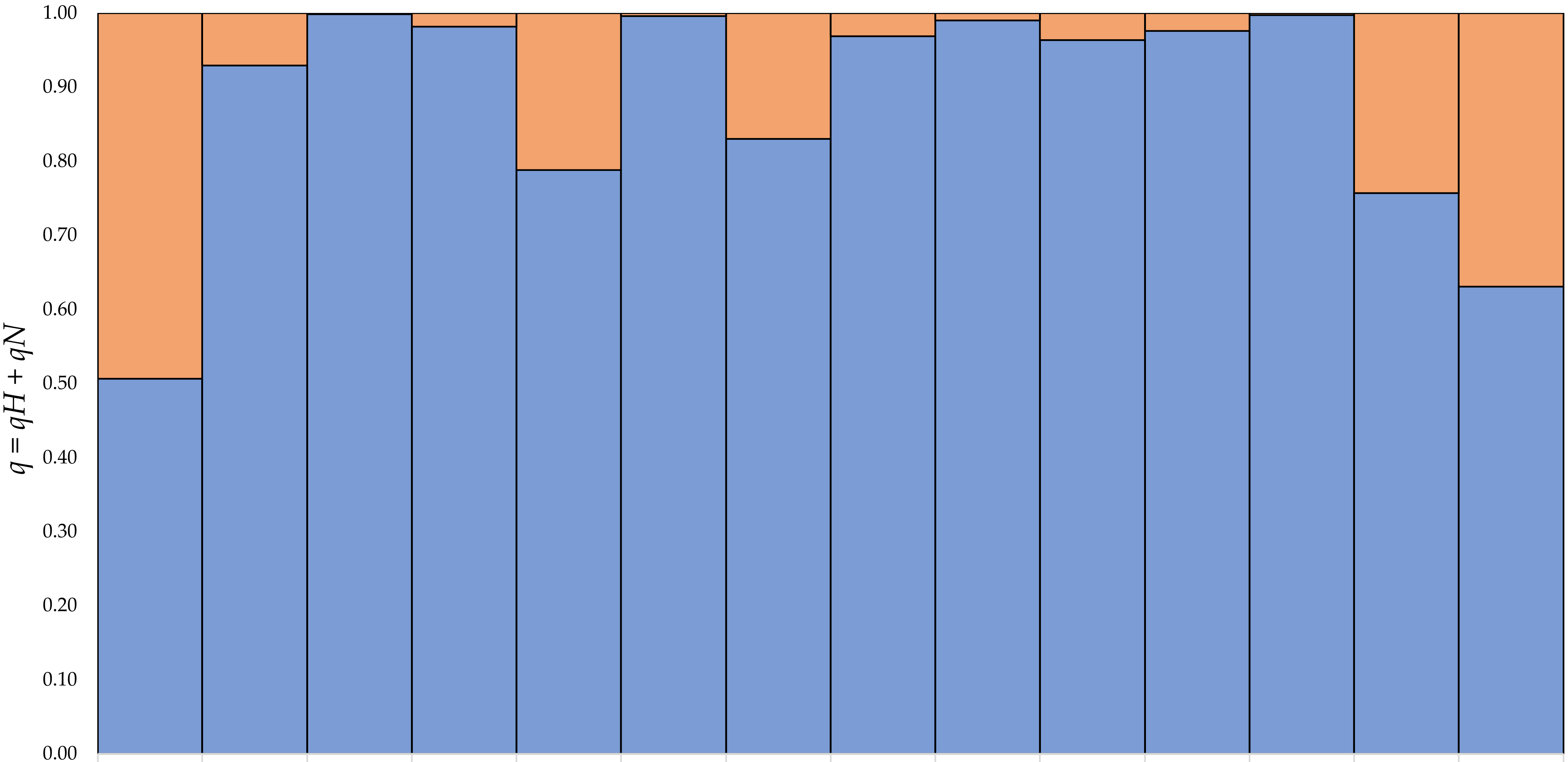

	496	497	498	499	500	501	502	503	504	505	506	507	508	509
qH	0.494	0.071	0.001	0.018	0.212	0.004	0.170	0.031	0.009	0.036	0.024	0.002	0.243	0.369
qN	0.506	0.929	0.999	0.982	0.788	0.996	0.830	0.969	0.991	0.964	0.976	0.998	0.757	0.631

CT17

Figure S8. STRUCTURE plot for the individual admixture coefficient values (q) at CT17 using the 19 diagnostic SNPs genotyped with MassARRAY approach. Each individual is represented as a vertical bar partitioned into segments according to the proportion of the genome belonging to each of the two clusters (K) identified (Hatchery (H) and Native (N), respectively) by STRUCTURE.

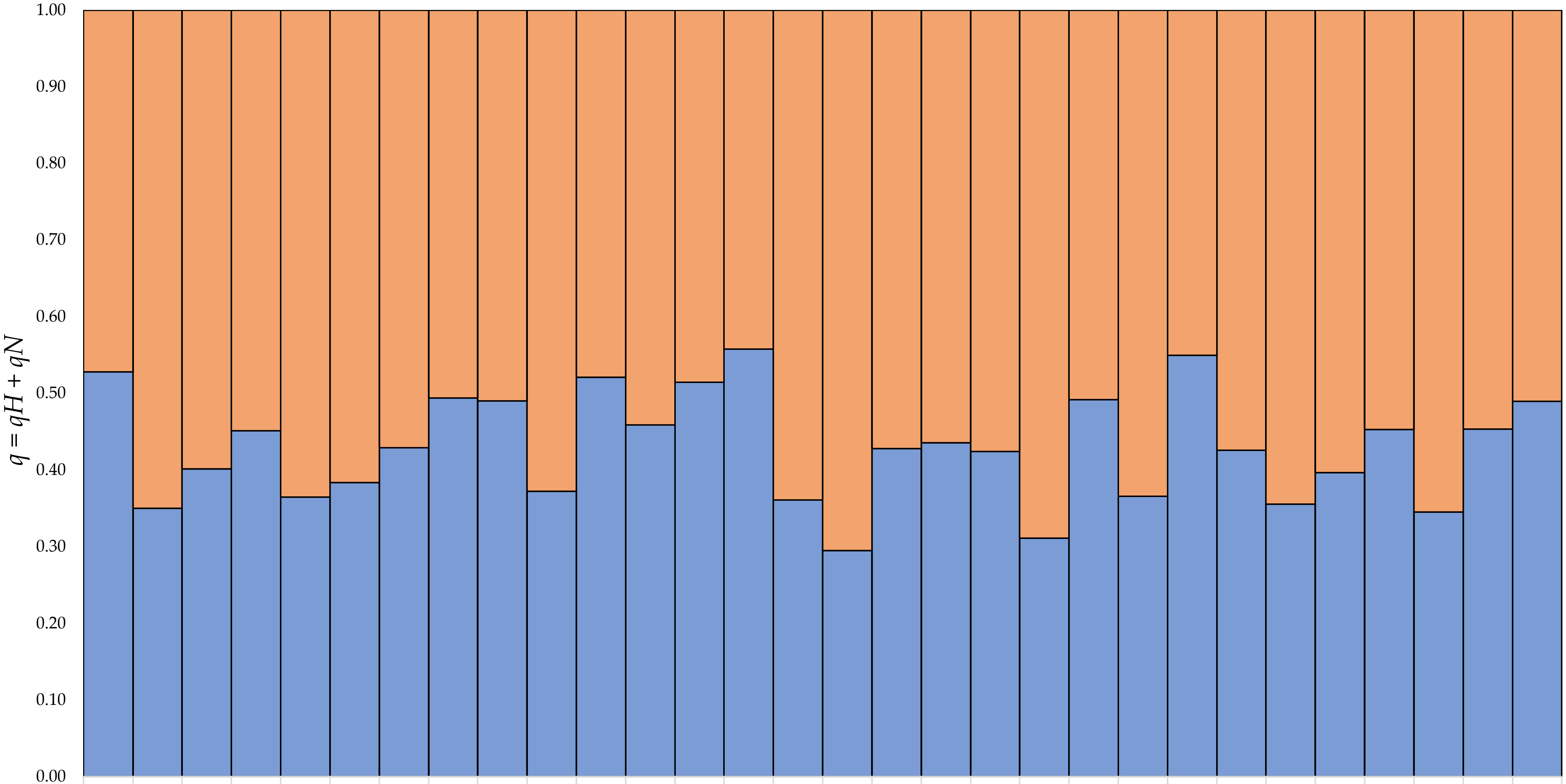

	206	207	208	209	210	211	212	213	214	215	216	217	218	219	220	221	222	223	224	225	226	227	228	229	230	231	232	233	234	235
qH	0.472	0.650	0.599	0.549	0.635	0.616	0.571	0.506	0.510	0.628	0.479	0.541	0.485	0.442	0.639	0.705	0.572	0.565	0.576	0.689	0.508	0.634	0.450	0.574	0.644	0.603	0.547	0.654	0.547	0.510
qN	0.528	0.350	0.401	0.451	0.365	0.384	0.429	0.494	0.490	0.372	0.521	0.459	0.515	0.558	0.361	0.295	0.428	0.435	0.424	0.311	0.492	0.366	0.550	0.426	0.356	0.397	0.453	0.346	0.453	0.490

GRE17

Figure S9. STRUCTURE plot for the individual admixture coefficient values (*q*) at GRE17 using the 19 diagnostic SNPs genotyped with MassARRAY approach. Each individual is represented as a vertical bar partitioned into segments according to the proportion of the genome belonging to each of the two clusters (K) identified (Hatchery (H) and Native (N), respectively) by STRUCTURE.

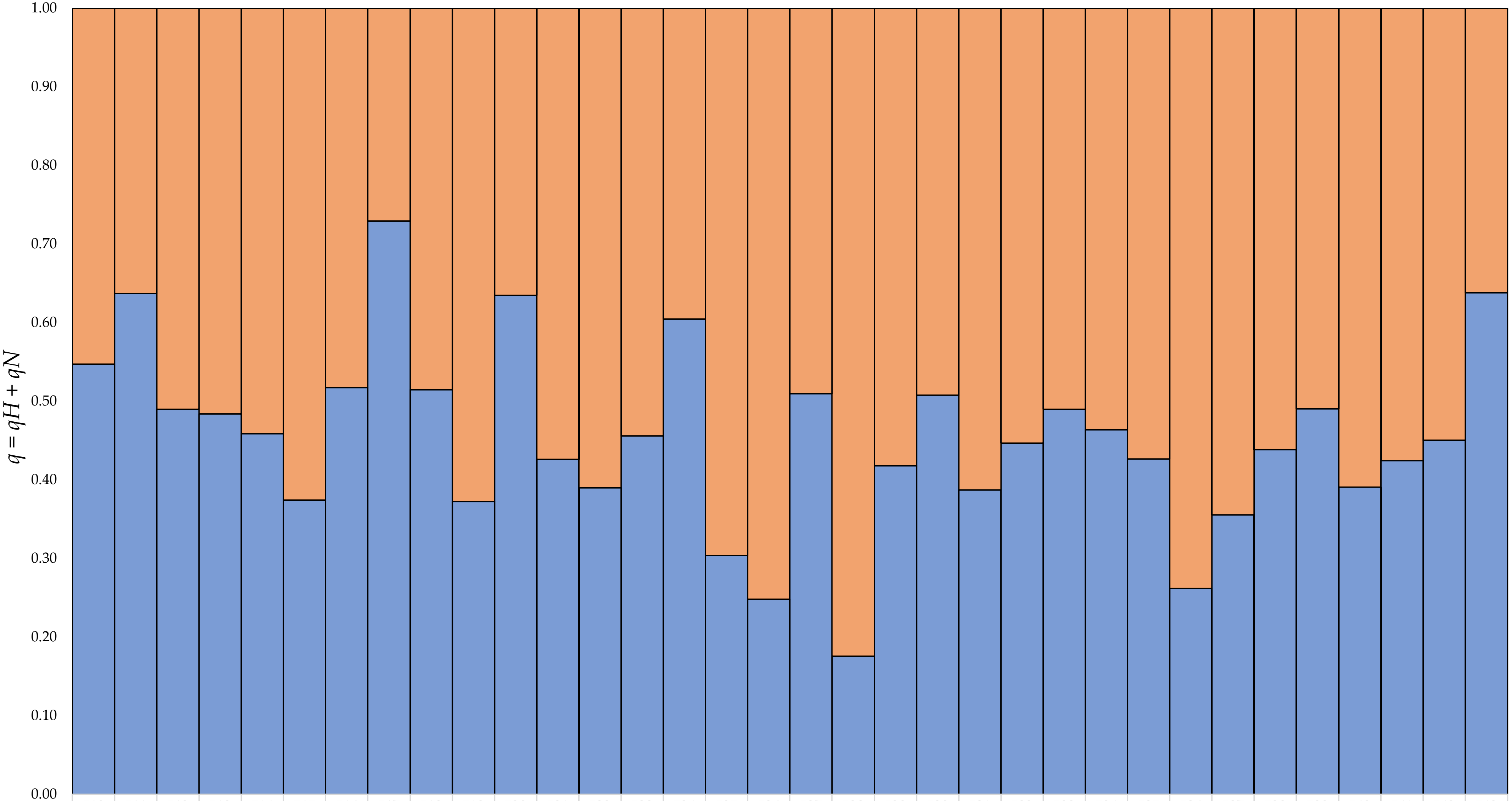

	510	511	512	513	514	515	516	517	518	519	520	521	522	523	524	525	526
qH	0.453	0.363	0.510	0.516	0.541	0.626	0.483	0.271	0.485	0.628	0.365	0.574	0.610	0.544	0.395	0.696	0.752
qN	0.547	0.637	0.490	0.484	0.459	0.374	0.517	0.730	0.515	0.372	0.635	0.426	0.390	0.456	0.605	0.304	0.248

	527	528	529	530	531	532	533	534	535	536	537	538	539	540	541	542	543
qH	0.490	0.824	0.582	0.492	0.613	0.553	0.510	0.536	0.573	0.738	0.645	0.561	0.510	0.609	0.576	0.550	0.362
qN	0.510	0.176	0.418	0.508	0.387	0.447	0.490	0.464	0.427	0.262	0.355	0.439	0.490	0.391	0.424	0.450	0.638

RT17

Figure S10. STRUCTURE plot for the individual admixture coefficient values (q) at RT17 using the 19 diagnostic SNPs genotyped with MassARRAY approach. Each individual is represented as a vertical bar partitioned into segments according to the proportion of the genome belonging to each of the two clusters (K) identified (Hatchery (H) and Native (N), respectively) by STRUCTURE.

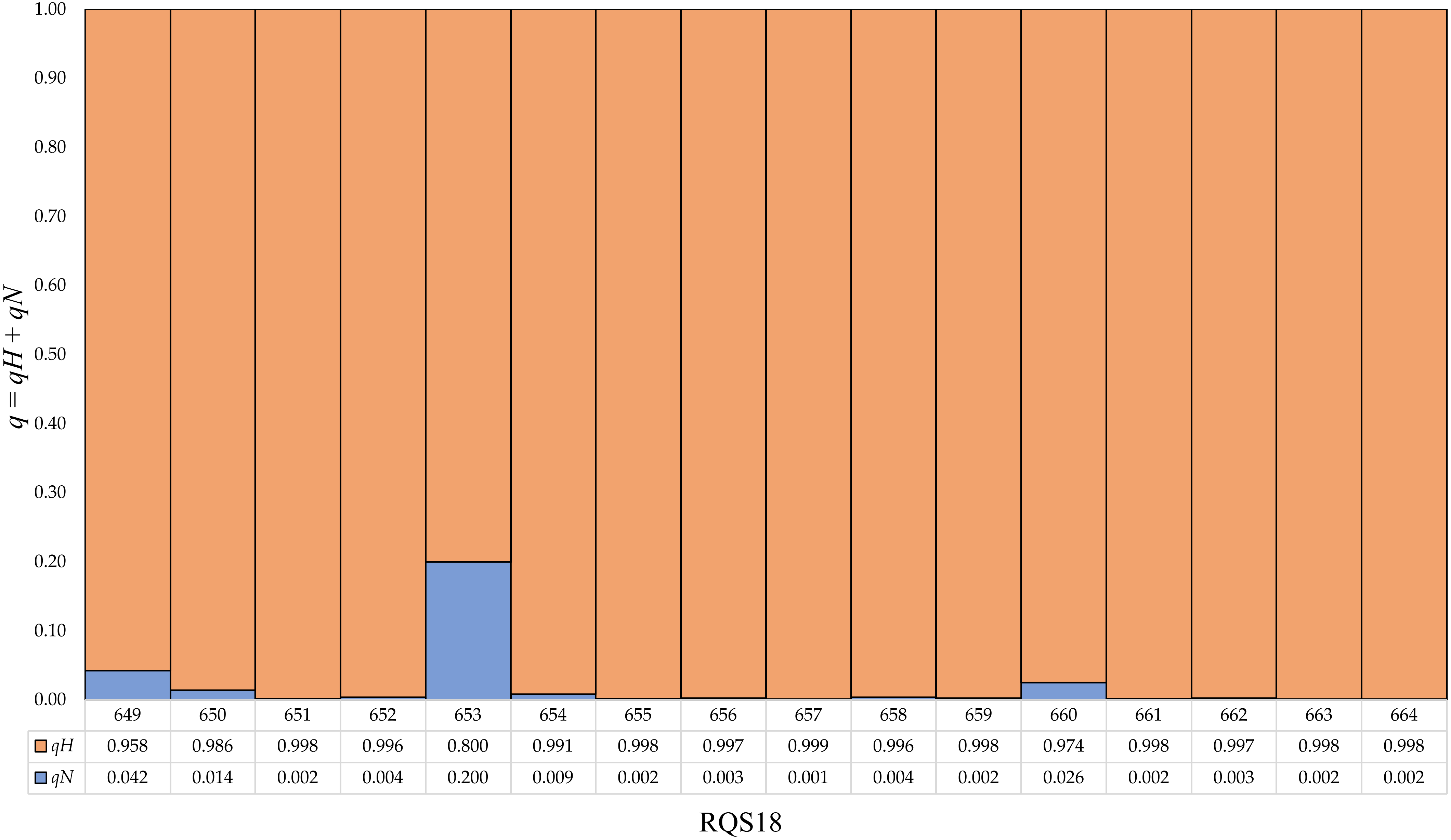

	649	650	651	652	653	654	655	656	657	658	659	660	661	662	663	664
qH	0.958	0.986	0.998	0.996	0.800	0.991	0.998	0.997	0.999	0.996	0.998	0.974	0.998	0.997	0.998	0.998
qN	0.042	0.014	0.002	0.004	0.200	0.009	0.002	0.003	0.001	0.004	0.002	0.026	0.002	0.003	0.002	0.002

Figure S11. STRUCTURE plot for the individual admixture coefficient values (*q*) at RQS18 using the 19 diagnostic SNPs genotyped with MassARRAY approach. Each individual is represented as a vertical bar partitioned into segments according to the proportion of the genome belonging to each of the two clusters (K) identified (Hatchery (H) and Native (N), respectively) by STRUCTURE.
