# Supplementary material for: Genomic Hatchery Introgression in Brown Trout (Salmo trutta L.): Development of a Diagnostic SNP Panel for Monitoring the Impacted Mediterranean Rivers
Source: Genes (Basel). 2022 Jan 28;13(2):255. doi: 10.3390/genes13020255 (PMC8872556; doi:10.3390/genes13020255)
Supplement: Supplementary file 1 [file genes-13-00255-s001.zip › SupMat_Casanovas/Text S1 Casanova et al. 2021 Genes.pdf]

Text S1. Information about main software parameters and command lines used in the sections *2.3. SNP calling and genotyping: defining a SNP panel for all locations studied* and *2.5. Multiplex MassARRAY design and implementation*.

Main options used for reads alignment and SNP genotyping:

Step	Software/module	Main options
Reference genome indexing and reads alignment	**Bowtie 1.3.0**	
	bowtie-build	—
	bowtie	--best --strata<br>-m 1<br>-v 3
SNP calling and genotyping	**Stacks 2.41**	
	gstacks	--model marukilow<br>--var-alpha 0.05<br>--gt-alpha 0.05<br>--max-clipped 0<br>--max-insert-len 0
	populations	—

Command lines:

2.3. SNP calling and genotyping: defining a SNP panel for all locations studied.

Bowtie 1.3.0

bowtie-build to index reference genomes

bowtie-build -f ReferenceGenome.fna Basename of the Bowtie index --threads 7 -q

Reads alignment against index (one command per sample)

bowtie --best --strata -m 1 -v 3 -p 7 --chunkmbs 20000 --sam -q --no-unal -x /Basename of the Bowtie index /sample.fq /sample.sam 2> /sample.log

Stacks 2.41

SNPs calling and genotyping

gstacks -t 7 -I /bams/ -O /gstacks/ -M /popmap.txt --model marukilow --var-alpha 0.05 --gt-alpha 0.05 --max-clipped 0 --max-insert-len 0

populations -t 7 -P /input/ -M /popmap.txt -O /output/ --vcf –genepop

2.5. *Multiplex MassARRAY design and implementation.*

Extract adjacent sequences to target position from reference genome (sequence length = 201 nucleotides)

perl FlankingRegions.pl -p /targetpositions.txt -g /ReferenceGenome.fna -o /output.fasta -s 100

Github link: https://github.com/adriancasanovachiclana/Genomic-scripts

Online manuals

Bowtie

http://bowtie-bio.sourceforge.net/manual.shtml

Stacks

http://catchenlab.life.illinois.edu/stacks/

References

Bowtie

Langmead, B.; Trapnell, C.; Pop, M.; Salzberg, S.L. Ultrafast and memory-efficient alignment of short DNA sequences to the human genome. *Genome Biol.* **2009**, *10*, R25, doi:10.1186/gb-2009-10-3-r25.

Stacks

Catchen, J.M.; Amores, A.; Hohenlohe, P.; Cresko, W.; Postlethwait, J.H. Stacks: Building and genotyping loci de novo from short-read sequences. *G3 Genes, Genomes, Genet.* **2011**, *1*, 171–182, doi:10.1534/g3.111.000240.

Catchen, J.; Hohenlohe, P.A.; Bassham, S.; Amores, A.; Cresko, W.A. Stacks: An analysis tool set for population genomics. *Mol. Ecol.* **2013**, *22*, 3124–3140, doi:10.1111/mec.12354.

Rochette, N.C.; Rivera-Colón, A.G.; Catchen, J.M. Stacks 2: Analytical methods for paired-end sequencing improve RADseq-based population genomics. *Mol. Ecol.* **2019**, *28*, 4737–4754, doi:10.1111/mec.15253.
